# Biology as involving laws and inconceivable without them

**DOI:** 10.1007/s12064-023-00384-2

**Published:** 2023-01-22

**Authors:** Richard Creath

**Affiliations:** School of Life Sciences, Arizona State University, 427 E. Tyler Mall, Tempe, AZ 85287-4501 USA

**Keywords:** Laws, History, Biology, Beatty, Windelband

## Abstract

There is an old attempt to divide the sciences into sciences of laws and the historical sciences. More recently, John Beatty has drawn the distinction so that biology is a historical science and urged that there are no genuinely biological laws. This paper shows that there are indeed biological laws, specifically statistical ones, notably in evolutionary theory. Moreover, all or almost all other areas of biology involve laws as well. Even history involves laws. Finally, the paper shows that this pervasiveness of laws is compatible with the most basic commitments of those who, like Beatty, would claim that biology is only historical.

## Introduction

My thesis is simple. I claim that there are laws in biology. In particular, there are laws in evolutionary theory. And, there are also genuinely biological laws throughout the discipline from cellular dynamics, to normal growth and development, and on to animal and even plant behavior. This is part of a larger story about the sciences, namely that all of them involve laws and all of them involve history (Cf., Creath 2010). I don’t know whether these claims ought to be controversial. But people I admire have, in the fairly recent past, said things that seem to reject my claim. I say “seem to reject” because I’m not sure how far the conflict extends. In fact, one can read some of these views in such a way that their authors’ core claims are both importantly right and fundamentally consistent with my remarks here.

In order to make my case, this essay divides into six parts: First, I will sketch a couple of views that divide scientific disciplines into those that aim at laws and those that aim at histories. Second, in order to make my claim clearer, I say just a bit more about how I am using certain terms. Third, I give an argument as to why evolutionary theory should be seen as involving at least one law. Fourth, I show why most other domains in biology should be thought of as also involving laws. Fifth, I show how laws are involved in any historical account we give. Finally, I reflect on whether there is any conflict between my position and that of those who have seemed to deny that there are laws in biology.

## Law governed versus historical

My story begins in 1894 when Wilhelm Windelband, in a famous and influential lecture (Windelband 1894/1980), distinguished between those empirical sciences that focused on laws exclusively and those that focused on history. He had no sooner made the distinction than he undermined it by admitting, in a passage that could have come straight out of Hempel, that one could have no explanations even in the historical sciences without laws (Hempel 1942). From our point of view, an important feature of Windelband’s own treatment of his distinction is that he clearly counted both biology and psychology as on the law-focused side rather than on the historical side.

About a hundred years after Windelband’s lecture, John Beatty famously employed this same distinction between sciences that are law focused and those that are historical (Beatty 1995). But, Beatty claimed that biology is a historical science and that biology has no laws of its own.^1^ Moreover, species are best understood as individuals. While there are clearly biological explanations, and explanations may require laws, it does not follow that those needed laws will be biological. The required laws could come from chemistry or physics or even geology. So according to Beatty, the story of evolution is a history of individuals. There are no laws of evolutionary history because that history is radically contingent. This contingency is made vivid by Gould’s image of replaying the tape of life (Gould 1989 p 48). Were one counterfactually to do so, it is virtually certain that the outcomes would be different.

There is much to agree with in Beatty’s view just sketched. Much of it is undoubtedly right. And much of the rest *may* be right. I don’t know. But there is at least the appearance of disagreement between Beatty’s claim that there are no laws in biology and my claim that there is at least one law in biology. To see whether there is a genuine disagreement behind the appearance of conflict, I will need to be a bit clearer on what I mean by such terms as “biology,” “laws,” and “evolutionary theory.” I won’t attempt anything like full definitions or specifications of these notions, but I need to be more specific than I have been so far. Only then can I indicate what sort of laws I think there are, where they are to be found, and why I think our biological theories commit us to such laws.^2^

I think that much in Beatty’s argument hinges on what one counts as genuinely biological. For example, if some portion of biology were reducible to chemistry, would the laws heretofore thought to be biological “really” be chemical or physical instead? I don’t want my thesis to depend either way on the reducibility of biology, so I will make no commitments on that. Even apart from reducibility, the issue is not always clear. The transformations described in gene regulatory network models strike me as both biological and law governed. But because these processes are at the molecular level, it *might* be claimed that the laws governing the transformations are chemical rather than biological. Disagreements over what is really biological are difficult to adjudicate because they are often struggles over the “soul” of biology, over what is important or promising. I think that philosophers should politely but firmly avoid taking sides on the soul of biology. In any case, I plan to sidestep the issue and choose an example from evolutionary theory. I take it that those who would insist that obviously molecular regularities are chemical or physical would agree that evolution is biological. So, if we can find a law here, it would be non-controversially biological.

## A bit more clarity on terms

To address the question of whether there are genuinely biological laws, we have to get clearer on what laws are and what it takes for one of them to be genuinely biological. I won’t attempt formal definitions. That is beyond my purpose at the moment. But, I will try to tell you a little more about what I mean. Laws are generalizations, of course—but which? The literature on laws is daunting. (Cf. (Reichenbach 1954/1976), (Hempel 1966), (Goodman 1955), and (Cartwright 1983)) We are told variously that.

- Laws are those generalizations that give necessary connections between the major terms of the generalization. This is promising but too strong in suggesting that the connection is invariable, thus ruling out statistical laws. Moreover, the exact meaning of “necessary” is a bit unclear, as is the source of our knowledge of that necessity.
- Laws are those generalizations that support counterfactual conditionals. (Goodman 1955 Chapter 1)
- Laws are those generalizations that can serve as the basis of explanations. (Hempel 1966 p 56)
- Laws are those generalizations that can be supported by their instances.

These are all plausible but hard to use. Accounts of counterfactual conditionals range from the dismissive to the further reaches of modal metaphysics. I would like to avoid both here. Both the second and fourth accounts involve the notion of support. Pending a detailed account of what can support what, these accounts of laws will have to remain rather informal.

Much of the discussion specifically of Beatty’s claim that there are no genuinely biological laws is directly about exceptionless universal generalizations that are true everywhere and everywhen. This would seem to sidestep statistical laws, that is, the generalization that say that within some group of entities or events a certain proportion have a given feature. This is important because the laws that I shall claim for evolutionary theory are in fact statistical. But this is not a disagreement between my position here and that of other main participants^3^ in the discussion of Beatty’s claim. All of them, so far as I know, agree that statistical laws are genuine laws. Presumably, if they did not specifically mention statistical laws, it was only because the theses and arguments they advanced did not require doing so.

It is sometimes urged as a necessary condition on laws rather than as a definition of them that laws must be purely qualitative, i.e., not involve essential reference to particular individuals. But as Nelson Goodman has shown (Goodman 1955 pp 79–80), the very notion of the purely qualitative still needs a lot of work before it can be used at all. And for the most part, references to individuals can be replaced with definite descriptions.

I would frame my notion of a law somewhat differently, though still informally as follows:

Laws are those generalizations that imply non-accidental (non-random) connections among particular events.

It is this that allows us to *use* laws to make inferences from some combination of events to some other event. Such a notion owes something to all three of the accounts given above and shares some of their liabilities as well. Whether the connections I speak of are merely verbal rather than “real” I simply won’t address here. And certainly at some point, I will have to rely on our ordinary judgments about what cases are non-accidental/non-random. Note that my account does not require laws to be true. There will of course be those who reject my use of the word “law” for this or some other reason. OK, call the notion I’ve sketched the lawlike generalizations. I’ll continue to use the word “law,” but substitute “lawlike generalization” if you prefer.

The idea that laws are those generalization that we can use to draw inferences among particular events is called a “pragmatic conception of laws” by Sandra Mitchell (1997). And as she points out this notion is far broader that the idea of exceptionless universal generalizations that are true everywhere and everywhen. The laws that I shall, in §3, are embedded in evolutionary theory fall within this conception, as do the examples of more specific laws pointed to in § 4.

So, when is one of these laws genuinely biological? *It’s when the events thus non-accidentally connected involve genuinely individuals, one or more place relations, or processes that are genuinely biological.* (Cf. Sober 1997) It makes no difference to my argument whether these connections are ultimately explainable in purely physico-chemical terms. At this level of organization, they are still biological events, individuals, relations, and processes. Perhaps, it will turn out to be true that I am nothing over and above a bag of chemicals. But even if that should turn out to be true, at this level of organization, I am still a biological entity, and the beating of my heart and the consequent circulation of my blood are still biological processes. No subsequent physio-chemical re-description of me will change that.

There is one more term that still needs to be clarified—at least a little. And that is “evolutionary theory.” Again, I will not try to define it precisely. There is already a sizable literature on this, and I doubt that anything I could say would improve on it. But I do mean something broader than natural selection or just changes in gene frequencies due to selective pressures. That would be compatible with there being a fixed stock of species when life began—a stock that gets differentially eroded via natural selection—but no new species. What makes Darwin’s theory exciting is that it is more than this. It is also a theory of the origin of new species and other taxa via the joint operation of natural selection and chance variation on heritable characteristics.

## Laws and evolutionary theory

So, chance variation is essential to Darwinian evolutionary theory, and it with this that we find laws, albeit statistical ones. If reproduction followed a universal law of exact replication, then there would be no novelty, no origin of species that is accounted for by evolutionary theory. So, what we need to capture this is a (statistical) law that can be stated rather informally as:

- In any reproductive event, there is a non-zero probability of variation among heritable features.

This is not exactly the same point that McShea and Brandon make in talking about “zero force evolutionary laws (2010),” but the two claims are alike in focusing on variation.

Even the law just stated is not enough. If the variations were utterly arbitrary and without limit, then there would be no heritability, no reproduction in the usual sense, and no enduring species at all. So, we need at a minimum to supplement the above law with this law:

- In any reproductive event, the probability of a major reproductively viable variation, i.e., one that affects features used in classifying the organism as of that species, is very much less than one.

Without such laws as these, Darwin’s evolutionary answer to the question of how new species can arise is literally inconceivable.

If evolutionary processes contain many such reproductive events (and they do!), then if the so-called tape of life were replayed, it would almost certainly have a radically different outcome. Sometimes, the intent of denying that there are biological laws is to reject the ancient idea that nature, and hence evolution, is aiming at producing a certain outcome, namely the perfect organism, humans. This ancient idea gets rejected equally forcefully on my claim that evolution involves statistical laws at every point.

Moreover, given that the tape of life plays out differently, it could happen that some of the current lower-level regularities we see might not appear. Some of those correlations might thereby depend, be contingent on, the evolutionary history that produced them. This is an important part of Beatty’s evolutionary contingency thesis. But that a current correlation might have been absent does not imply that the now correlated features do not now have a common cause. We now quite properly *use* those correlations to guide our expectations. (Cf. Mitchell 1997) The correlation need not be accidental or wholly random. They are still, in the required sense, lawlike.

## Genuinely biological laws are everywhere in biology

Even if my argument thus far is successful, it does not yet show that laws are involved throughout biology. Let’s turn to that. Laws are sometimes said to be of two kinds: Laws of succession (what follows what) and laws of coincidence (what co-occurs with what), and the former are more basic. Consider the following case that exhibits a law of coincidence:

If an organism is a large (about 3 m nose to tail tip) tawny animal, feline in appearance, then it is probably an African lion (*Panthera leo*), and if it has an enormous mane, then the identification of species is even more certain. Having these features of mane, size, color, and shape is non-accidentally correlated with other features. Probably, it is also male and warm blooded, has a tuft of fur on the end of its tail, and behaves, for the most part, like a lazy oaf.

Even if you think that species are individuals and that this matters to whether one is dealing with laws, it makes no difference in this case. Here, the reference to *Panthera leo* is dispensable, and the remaining features are purely qualitative, i.e., make no essential reference to individuals. And they are still non-accidentally correlated. For the most part, combinations of qualities that go toward identifying an organism as of some biological kind are correlated not entirely accidentally. Generally, if an organism has a lot of the identifying features of a given kind, there is a good chance that it will have the others. This statistical correlation is supportable by instances. And the correlated features might have a common genetic cause.

Now it is possible to argue that from some cosmic point of view the correlation of identifying features really is accidental. But I’m not sure that I understand that point of view or whether *all* the laws of nature might be accidental from such a cosmic remove. But I doubt that there is any way of resolving this issue. The alternative sides may simply be using words in different ways. In any case, we may have reached the useful limits of our informal notion of non-accidental connections.

An even richer fund of laws in biology are the laws of succession. And where would we find those laws? In biological dispositions (behavior in the broad sense) and in biological processes. Among the latter are:

- Cell and embryonic development
- Normal growth, reproduction, and senescence in both animals and plants

The individuals involved are clearly biological, and the various stages of these processes succeed each other in plainly non-accidental, though statistical, ways.

Among the biological dispositions would be:

- All animal behavior: Poke a bull and he will attempt to poke you back.
- All plant behavior: Sunflowers follow the sun, and you can affect the behavior by intervening in various ways.

Are these laws biological? Yes, the phenomena thus connected are biological ones. Remember that I am here agnostic about whether any of these laws can be reduced to physics and chemistry. It makes no difference to the present argument. Even if they are reducible, laws governing organismal behavior and processes remain biological at this level because the organisms and processes themselves are biological.

## Laws and history

So, is biology a science of laws? Yes, every science is. Is biology historical? Yes, again, every science is. (Creath 2010) But biology, evolutionary biology, is historical in the sense that it presents the deep history of biological phenomena. But even this involves lawlike claims by presupposing biological laws governing the various events it presents. Even Windelband saw that there are always laws, non-accidental connections, behind the narratives of history.

The historical succession of events is not just one damn thing after another. The histories we write are not just lists of all the things that happened not even during short intervals. First, they are selections from among the events that we think happened. It is not possible to present them all. And second, they are narratives, that is, connected threads of events, albeit rather complex threads at times. What we say comes earlier should somehow be relevant to the occurrence of what we say comes later. If it’s irrelevant, it shouldn’t be there—except for, perhaps, to make the narrative more colorful. In our evolutionary stories, the tree of life is part of the narrative that includes the changing context in which these events take place. The events are biological and they have to be connected non-accidentally. If they weren’t connected in such lawlike ways, we would have only a list and no history.

The point extends to recent human history including the history of science. We need a selection and a narrative, that is, a connected thread of events. The historian need not and generally will not state the laws. But, in these areas, the historian needs enough of an understanding of how humans are disposed think and act, i.e., the non-accidental patterns of their behavior, to recognize the connectedness of the historical narrative. And the reader too has to see the connectedness of the events. There may be some “brute” facts in the historical narrative, the ones that prior events do nothing to help us understand. But if all events were brute in this way, then we would have no narrative; again, we would have only a list.

Can the events described in histories that make such tacit appeal to this sort of connectedness be contingent? Of course. Wherever the lawlike generalizations involved are statistical, and in biology and in human affairs they almost all are, the resultant sequence of events will be contingent given whatever stating point you choose, from whatever point you choose to replay the tape of life.

## How much conflict?

And this is where we started: With Windelband’s observation that even the “historical sciences” involve laws and with what I take to be John Beatty’s primary motivations—that evolutionary development of life proceeds contingently, that replaying the tape of life would probably yield a quite different outcome, and that biology can proceed without becoming a wholly owned subsidiary of physics or chemistry. With all of that I have agreed.

So how much disagreement is there? Well, I have argued first that there really are laws of (evolutionary) biology. I suppose that this does contradict Beatty’s stated position. But I think that there is rather less conflict with his main motivations. I argued further that notions of laws and the genuinely biological can be sketched according to which there are genuinely biological laws or lawlike claims to be found almost everywhere in biology, including in its straightforwardly historical parts. This broad extension of the lawlike into almost all of biology may seem even more decisively to contradict Beatty’s thesis that there are no biological laws.

Again, I suspect that here the disagreement is more apparent than real. First, Beatty was concerned to emphasize the importance of history for biology, in contradistinction to a certain philosophical conception of the physical sciences according to which they, or important parts of them, have no historical concerns at all. I would second Beatty’s emphasis on the historical character of biology and insist only that every other science is historical as well. (Creath 2010) Second, often talk of laws is about universal rather than statistical laws. I have not claimed that there are any laws of that universal kind in biology, so to that extent, Beatty and I do not disagree. And third, I am inclined to think that Beatty’s primary concern in rejecting laws governing evolution is to show that here are no laws governing the long-run *direction* of evolution, no laws telling us what its outcome must be. Evolutionary history is radically contingent. This is what the image of replaying the tape of life is all about. With this I agree. Beatty’s claim about contingency and my claim about statistical laws that are essential to evolutionary theory are not identical, but they are perfectly compatible. And there is considerable overlap. That the laws are statistical is what makes the radical contingency of evolutionary history possible, even virtually unavoidable.

Moreover, on the view that there are statistical laws among the fundamental laws of evolution, it can turn out that on a different evolutionary trajectory some of the lower-level regularities we currently see would not appear. Such regularities are, in Beatty’s sense, contingent. But as we saw, the now-correlated features might still have a common cause, and thus the correlation would be neither accidental nor wholly random. They can still be lawlike.

At last, we can return to Windelband. What he had to say was interestingly wrong (and that is a high compliment). My main quarrel is not with the idea that biology is historical but with the impulse to divide the sciences into radically different sorts. Of course, there are differences among the sciences. And there are differences within each science as well, and the character of each of the sciences continues to change at least in detail. But, there is no vast cleavage either in character or in method with respect to which the sciences fall neatly on one side or the other. So, I reject the very distinction he drew between two fundamentally different kinds of empirical science: Those sciences that seek laws but have no historical concerns and those that are historical to the exclusion of laws. In arguing that there are laws as part of evolutionary theory and indeed across biology, my point is not about where to draw Windelband’s distinction but about whether to draw that distinction at all.

## Footnotes

1 In a related discussion, Carol Cleland (2002) draws a contrast between historical science and experimental science. She quite rightly points out that the practices of historical and experimental scientists differ because their epistemological situations differ. She does not, however, claim that all and only experimental sciences have laws. So, her distinction is not quite the same as Beatty’s, and her argument would not establish the conclusion he is trying to draw. See also §5 below for a further discussion of whether there are sciences that are wholly historical or ever those that are wholly without it.

2 For other discussions of Beatty’s claim, see footnote 4.

3 For example, Beatty 1995, 1997, 2006, Carrier 1995, Sober 1993, 1997, Brandon 1997, 2006, Mitchell 1997 and Waters 1998. But see also Cleland 2002, Elgin 2006, DesAutels 2010, Elgin and Sober unpublished, and McShea, Wang, and Brandon 2019.
